# Coinoculation of *Pantoea phytobeneficialis* PF55 and *Trichoderma harzianum* ESALQ 1306 enhances soil microbial activity and agronomic performance in onion

**DOI:** 10.1007/s00203-026-05069-0

**Published:** 2026-07-21

**Authors:** Juliano Silveira Machado, Edenilson Meyer, Lázaro Nanque, Kenji da Cruz Konno, Kéllin Cristian Ribas Martins, Jorge Andres Betancur Gonzalez, Admir José Giachini, André Ricardo Zeist

**Affiliations:** 1 Department of Crop Science, Center for Agricultural Sciences, Federal University of Santa Catarina, João David Ferreira Lima Campus, Florianópolis, SC Brazil; 2 Department of Microbiology, Immunology and Parasitology, Center for Biological Sciences, Federal University of Santa Catarina, João David Ferreira Lima Campus, Florianópolis, SC Brazil; 3 Department of Crop Science, Luiz de Queiroz College of Agriculture, University of São Paulo, Piracicaba, SP Brazil

**Keywords:** Nutrient uptake, Sustainable agriculture, Plant growth-promoting microorganisms

## Abstract

Coinoculation with growth-promoting microorganisms has proven to be an efficient strategy for enhancing the agronomic performance of onion (*Allium cepa* L.). This study evaluated the effect of inoculating *Pantoea phytobeneficialis* PF55 and *Trichoderma harzianum* ESALQ 1306, applied individually and in combination, on agronomic, biochemical, and microbiological variables of the Valessul cultivar. The experiment was conducted in a randomized block design with four treatments (non-inoculated, PF55, ESALQ 1306, and PF55 + ESALQ 1306) and 12 replications in Florianópolis, SC, Brazil. Inoculation was performed 14 days after seedling transplant. Inoculation with PF55 resulted in a measurable increase in productivity. The results indicate that PF55 application increased productivity, biomass, and nutrient accumulation, while coinoculation with *T. harzianum* ESALQ 1306 promoted specific effects, such as higher carotenoid contents and soil microbial activity. Isolated inoculation with *T. harzianum* ESALQ 1306 increased chlorophyll b and total contents, as well as soluble solids levels. These results confirm that the bacterium acts as the primary growth-promoting agent, while the fungus plays a modulating role, influencing physiological and edaphic processes in a more restricted manner.

## Introduction

Onion (*Allium cepa* L.) is a vegetable of high nutritional value, rich in antioxidants, minerals, and other compounds that aid in disease prevention (Gupta et al. 2025). Advances in cultivation technologies and post-harvest management have contributed to increasing bulb productivity and quality (Altintas and Bal 2008; Younes et al. 2023). National production has gained increasing relevance, with average onion productivity in Brazil in 2023 estimated at 33,226 kg ha⁻¹, highlighting the states of Santa Catarina, Goiás, and Bahia as production leaders (IBGE 2023). Between 2021 and 2022, production value increased by more than 60% (IBGE 2023), underscoring the economic and social importance of the crop. Despite this progress, observed average yields are still below the maximum potential seen in controlled experiments (Younes et al. 2023; Contreras-Cornejo et al. 2009; Altintas and Bal 2008), highlighting the need for biological technologies that reduce dependence on chemical inputs and enhance cultivation sustainability.

The use of bioinputs has expanded in different regions of Brazil. Various studies have demonstrated that these can increase plant growth and promote disease control (Minchev et al. 2021; Wang and Kuzyakov 2024). The use of bioinputs in onion cultivation represents a sustainable alternative to reduce dependence on chemical inputs and improve productive efficiency (Sarma et al. 2025; Younes et al. 2023). Growth-promoting microorganisms, such as beneficial bacteria and fungi, can favor plant development and contribute to the biological control of phytopathogens (Camacho-Luna et al. 2023; Nascimento et al. 2020). Thus, integrating bioinputs into onion management strengthens bulb productivity and quality, while promoting greater biological balance in the soil and sustainability in the cultivation system.

The application of fungi from the genus *Trichoderma* in onion cultivation has shown positive effects, such as reducing the use of chemical inputs and increasing productivity (Camacho-Luna et al. 2023). Species like *T. harzianum* and *T. asperellum* promote onion seedling growth, increasing dry mass and root length (Liriano et al., 2015). Coinoculation of *T. harzianum* with other beneficial microorganisms, associated with the addition of organic material, also yields positive results (Metwally et al. 2021). However, although the role of these fungi as biofungicides and growth promoters is consolidated in various crops (Sarma et al. 2025; Baek et al. 2025), the mechanisms of interaction with growth-promoting bacteria are not fully understood in onion cultivation. This gap is relevant because coinoculation can generate distinct effects, varying between synergism and competition depending on environmental conditions (Din et al. 2025). Thus, understanding how isolates interact with each other and with the plant is essential to advance the development of more effective and stable bioinoculants and management plans.

Soil biological activity refers to the set of processes carried out by living organisms in the soil, such as microorganisms and soil fauna, which affect essential functions like organic matter decomposition, nutrient cycling, respiration, enzyme production, and others. Soil enzymes, such as ureases, phosphatases, and β-glucosidases, are widely recognized as indicators of soil biological quality, as their activities are essential for maintaining the functionality and sustainability of the edaphic ecosystem (Adetunji et al. 2017). Additionally, soil biological activity can vary according to land use and management, influencing both the quantity of enzymes present and the microbiota composition (Mir et al. 2023). Furthermore, this biological activity is affected by factors such as soil type, cultivated plants, and management practices (Wang and Kuzyakov 2024; Mir et al. 2023), making it difficult to maintain at ideal levels. In this context, adopting strategies that favor microbial activity, such as the use of biological inoculants, becomes fundamental to balance the edaphic system and reduce dependence on chemical fertilizers. Agricultural intensification, with conventional management practices, often leads to simplification of soil microbiota and loss of ecological functions, limiting productive potential in the medium and long term. This scenario is particularly relevant for onion cultivation, which is mostly conducted in conventional systems highly dependent on external inputs, reinforcing the need for biological alternatives that improve soil quality and cultivation sustainability. Thus, stimulating microbial communities through the introduction of growth-promoting microorganisms can be a viable alternative to maintain biological fertility and ensure the sustainability of onion cultivation.

Fungi of the genus *Trichoderma* spp. are widely used in Brazil as biofungicides in crops such as soybean, cotton, corn, beans, strawberry, citrus, sugarcane, coffee, tobacco, vegetables, fruits, forests, and ornamentals, both in organic and conventional systems (Meyer et al. 2019). In addition to pathogen control, these fungi promote plant growth through the production of siderophores (Li et al., 2017), nutrient solubilization (Bedine et al. 2022), the production of ACC deaminase (Zhang et al. 2015), and the synthesis of auxins and gibberellins (Contreras-Cornejo et al. 2025). This genus can act as endophytic, saprophytic, or mycoparasitic, interacting with microorganisms, plants, and arthropods (Macías-Rodríguez et al. 2020). This functional versatility makes it one of the most studied genera in agricultural biotechnology, as it combines biocontrol and growth promotion effects in a single organism (Camacho-Luna et al. 2023; Liriano et al., 2015; Yağmur et al. 2024). Although the potential of these fungi is widely recognized in different crops, the response can vary depending on the cultivated species, the isolate used, and edaphoclimatic conditions, making specific studies necessary for each productive system (Asghar et al. 2024). Some species of this genus can increase onion bulb growth (Younes et al. 2023), while others, such as *T. longibrachiatum* and *T. asperellum*, induce resistance against *Fusarium oxysporum* f. sp. cepae and *Sclerotium cepivorum*, respectively (Abdelrahman et al. 2016; Rivera-Méndez et al. 2020). *T. atroviride* promotes root growth and nutrient absorption (Colla et al. 2015).

Growth-promoting bacteria show potential in onion cultivation, increasing growth and yield (Younes et al. 2023; Singh et al. 2019; Severo et al. 2025). *Pantoea* species exhibit beneficial interactions with plants (Doni et al. 2021), with the strain *Pantoea phytobeneficialis* PF55 promoting growth via nitrogen fixation, phosphate solubilization, hormone production, and siderophore synthesis (Nascimento et al. 2020). This evidence suggests that both fungi of the genus Trichoderma and bacteria such as *P. phytobeneficialis* PF55 can play complementary roles in onion development. However, most studies evaluated these microorganisms individually, without considering possible interactions. This gap is particularly relevant given the challenges of onion production systems, characterized by high demand for fertilizers, intensive pesticide use, and reduction in soil microbial biodiversity in successive crops. Thus, coinoculation of compatible microorganisms emerges as a promising alternative to increase productivity, modulate physiological characteristics, and stimulate soil microbial activity, promoting more sustainable cultivation systems.

Given this context, this study aimed to evaluate the effect of inoculating *P. phytobeneficialis* PF55, individually or in coinoculation with *T. harzianum* ESALQ 1306, on productivity, biochemical characteristics, and soil microbial activity in onion plants of the Valessul cultivar.

## Methods

### Experimental location

The experiment was conducted at the Ressacada Experimental Farm of the Federal University of Santa Catarina, in Florianópolis, SC, Brazil, at coordinates 27° 41’ 06.28” S; 48° 32’ 38.81” W, between August 27 and December 5, 2024. The local soil is classified as Typic Quartzarenic Hydromorphic Neosol, according to the Brazilian Soil Classification System (EMBRAPA, 2018). The inicial characteristics of the 0–20 cm soil layer include: pH H_2_O = 5.5; organic matter = 47 g kg⁻¹; P (Mehlich^− 1^) = 2.5 mg dm⁻³; exchangeable Ca = 2.9 cmol_c dm⁻³; exchangeable Mg = 2.5 cmol_c dm⁻³; exchangeable K = 72.7 mg dm⁻³; CEC = 10.12 cmol_c dm⁻³; V% = 54.5. According to the Köppen climate classification, the climate is Cfa subtropical, constantly humid, without a dry season, with hot summers and an average annual precipitation of 1,500 mm (INMET 2017).

### Treatments

The experiment evaluated the effect of inoculating *P. phytobeneficialis* PF55, individually or in coinoculation with *T. harzianum* ESALQ 1306, on the productivity and biochemical characteristics of onion, Valessul cultivar. Four treatments were tested: (1) non-inoculated control (NI); (2) PF55; (3) ESALQ 1306; and (4) PF55 + ESALQ 1306.

### Installation and experimental management

The experiment was conducted in a direct planting system over previously established white oat straw. Initially, the experimental area underwent soil acidity correction with 500 kg ha⁻¹ of lime, followed by mechanical preparation restricted to bed formation, ensuring structural uniformity of the area before oat planting. For straw formation, 45 kg ha⁻¹ of NPK 5–25-25 and 2 kg ha⁻¹ of manganese sulfate were applied during oat development.

The experimental design was randomized blocks, with four treatments and twelve replications, totaling 48 plots. Each plot measured 2.0 × 1.4 m, containing five rows spaced 25 cm apart and 10 cm between plants, totaling 100 useful plants per experimental unit. The cultivar used was SCS373 Valessul, transplanted with homogeneous vigor and vegetative pattern. Onion seedlings were initially produced in polystyrene trays with 18 mL cells, containing commercial substrate Carolina Soil^®^, remaining in the nursery for 60 days. At the time of transplant, the seedlings had approximately 15 cm in height, well-formed pseudostem, and 5 to 6 true leaves, characterizing an adequate stage for field establishment.

At onion planting, 320 kg ha⁻¹ of NPK 5–25-25 and 40 kg ha⁻¹ of KCl were applied according to the official recommendation. Triple superphosphate was applied at 700 kg ha⁻¹, corresponding to 70% of the recommended rate, to establish a condition of moderate phosphorus availability. This strategy was adopted to establish a condition of moderate phosphorus availability, since phosphorus availability is known to influence the activity of phosphorus-solubilizing microorganisms and related enzymatic processes (Santos et al. 2022). Nitrogen fertilization followed the official recommendation through the NPK fertilizer at planting and urea applications during topdressing. Topdressing fertilization was split into two applications, totaling 85 kg ha⁻¹ of urea and 35 kg ha⁻¹ of KCl in the first stage, and 70 kg ha⁻¹ of urea and 35 kg ha⁻¹ of KCl in the second, according to vegetative growth and the onset of bulb development.

Weed management was performed with Clethodim (Select^®^) applied post-emergence. Insect control included Zeta-Cypermethrin (Mustang^®^), applied according to technical recommendations. Irrigation was conducted by sprinklers, maintaining adequate humidity for crop development throughout the cycle.

After complete seedling establishment, 14 days after transplant, inoculation with PF55 and ESALQ 1306 was performed to promote root colonization and plant–microorganism interaction. For treatments with *Trichoderma harzianum* ESALQ 1306, the commercial product TRICHODERMIL^®^ (guarantee of 2.0 × 10⁹ viable conidia mL⁻¹) was used at a dose of 1 L ha⁻¹. For treatments with the *Pantoea phytobeneficialis* PF55 strain, the commercial product BENEFARM PF55^®^ (guarantee of 1 × 10⁸ CFU mL⁻¹) was applied at a dose of 200 mL ha⁻¹. Each inoculant was diluted separately in sterile water according to the amount required per plot (2.8 m², containing 100 plants) to a final volume of 200 mL. In the coinoculation treatment, the inoculants were applied sequentially, one immediately after the other, using separate suspensions. The suspensions were uniformly distributed over the entire plot by applying them along the planting rows using a garden watering can. A planting density of 300,000 plants ha⁻¹ was considered.

### Evaluated characteristics

1. Soil enzymatic activity

Soil samples were collected 60 days after inoculation (74 days after transplant). Subsequently, laboratory analyses were conducted.

To assess fluorescein diacetate (FDA) hydrolysis, 1.0 g of soil was incubated for 2 h at 25 °C with 0.1 mL of a 4.8 mmol L⁻¹ FDA solution and 19.9 mL of a 60 mmol L⁻¹ sodium phosphate buffer at pH 7.6. The reaction was terminated by adding 20 mL of acetone, followed by centrifugation of the mixture at 4,000 rpm for 5 min. The supernatant was then filtered, and absorbance of the extract was read at 490 nm (Alef and Nannipieri 1995).

2. Yield and growth parameters

Bulb harvest was initiated when about 80% of the plants reached the “snap” stage. After harvest, plants were field-cured and subsequently kept in a protected environment until the start of laboratory analyses.

Productivity was obtained by weighing all useful bulbs from each plot, with subsequent conversion to t ha⁻¹. Then, a sample of 15 bulbs per plot was separated for quality analyses, ensuring standardization between treatments. Other samples of 15 bulbs were destined for dry mass determination and nutrient analyses.

Bulb water content, root dry mass, and shoot dry mass were determined by drying samples in a forced-air oven at 60–65 °C until constant mass.

3. Bulb quality

For soluble solids, titratable acidity, and pH, fifteen bulbs per plot were crushed and filtered. Soluble solids content (°Brix) was determined with a refractometer. Titratable acidity, expressed as % pyruvic acid, was obtained by titrating 20 mL of extract diluted in 100 mL of distilled water, plus 0.3 mL of phenolphthalein, with 0.1 N NaOH until color change. pH was determined with a benchtop pH meter.

4. Photosynthetic pigments

Shoot samples for pigment analyses were collected before the snap stage, 50 days after transplant.

For photosynthetic pigments, leaf fragments (1.41 cm²) were immersed in 3 mL of dimethyl sulfoxide (DMSO) and incubated for 48 h at 25 °C. An aliquot of 0.2 mL was transferred to microplates, and absorbance was measured at 480, 649, and 665 nm. Contents of chlorophylls *a*, *b*, and carotenoids were calculated according to Wellburn (1994).

5. Phosphorus and potassium contents

Shoot samples for nutrient analyses were collected 51 days after transplant, whereas roots and bulbs were collected at harvest.

Phosphorus and potassium analyses were performed separately in the shoot, bulbs, and roots. Plant tissues were washed, dried in an oven, and ground to obtain homogeneous samples. Nutrient determination followed Tedesco et al. (1995), with acid digestion and subsequent instrumental reading. Phosphorus (P) was quantified by spectrophotometry at 660 nm, while potassium (K) was determined by flame photometry.

### Statistical analyses

Data were subjected to normality tests (Shapiro-Wilk) and homogeneity of variances (Levene), followed by analysis of variance (ANOVA) and Tukey’s test (*p* < 0.05). Analyses were performed in R software v. 4.3.1 (R Core Team 2022), with the AgroR package (Shimizu et al. 2023), considering the randomized block design and the factorial experiment.

## Results

**Fig. 1:**
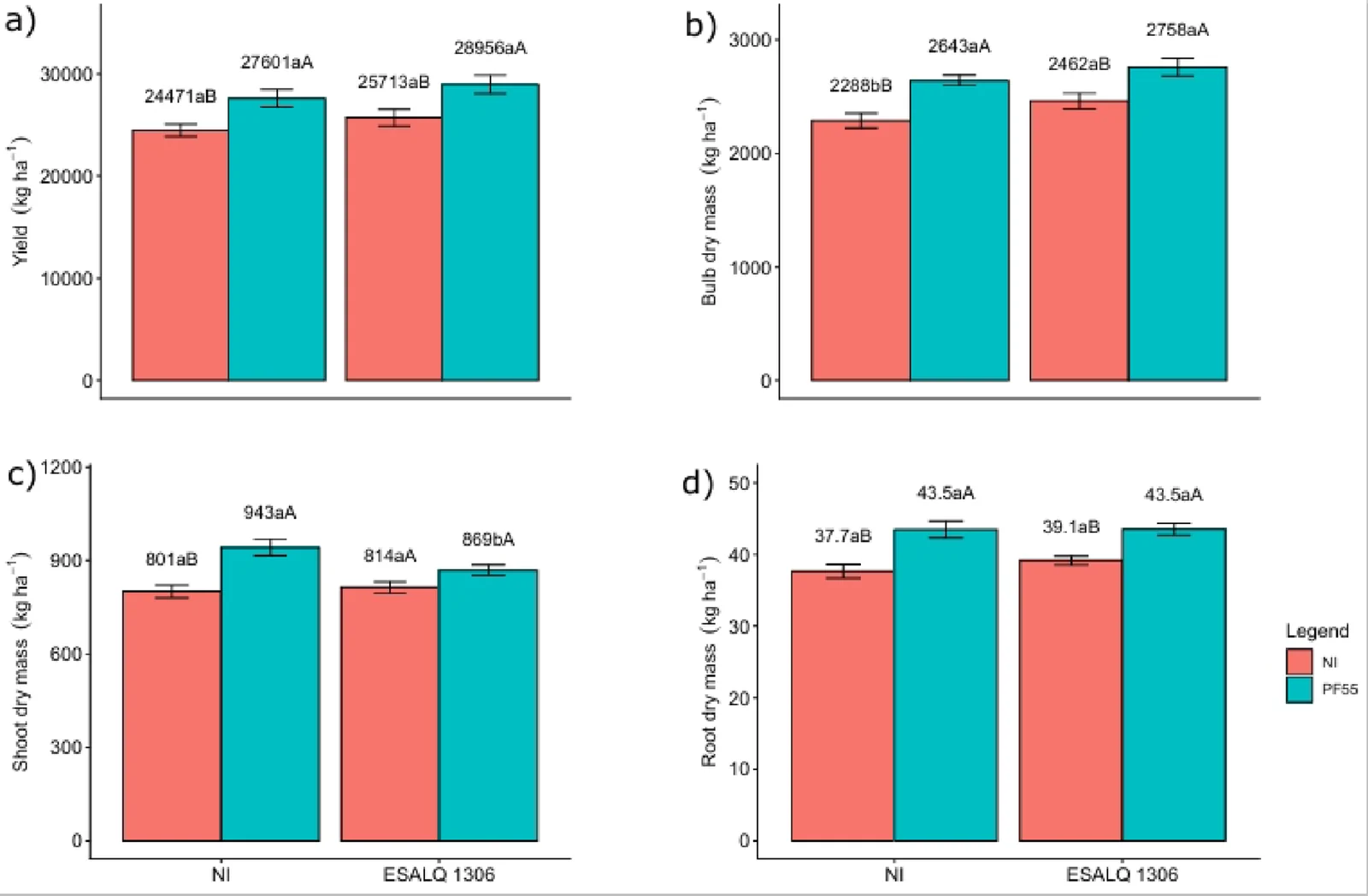
Yield (**a**), bulb dry mass (BDM) (**b**), shoot dry mass (SDM) (**c**), and root dry mass (RDM) (**d**) of onion cultivar Valessul under inoculation with PF55 and ESALQ 1306. Bars represent means ± standard error. Red and blue bars represent the absence (NI) and presence (PF55) of bacterial inoculation, respectively. Different lowercase letters indicate significant differences between ESALQ 1306 levels within each PF55 level (same bar color), whereas different uppercase letters indicate significant differences between PF55 levels within each ESALQ 1306 level (adjacent bars), according to Tukey’s test (*p* < 0.05)

Inoculation with PF55 increased onion yield by approximately 12.8% compared to treatments without the bacterium (*p* < 0.05), regardless of ESALQ 1306 presence (Fig. 1a). For bulb dry mass, PF55 inoculation resulted in an increase of nearly 15.5% relative to the control (*p* < 0.05), while isolated ESALQ 1306 showed an intermediate effect (Fig. 1b). Shoot dry mass was higher only when PF55 was applied alone, representing an increase of approximately 17.7% relative to the control, with no difference in coinoculation (Fig. 1c). Root dry mass was also favored by PF55, with gains of 17.7% and 11.3% compared to the non-inoculated control and isolated ESALQ 1306, respectively (Fig. 1d).

**Fig. 2:**
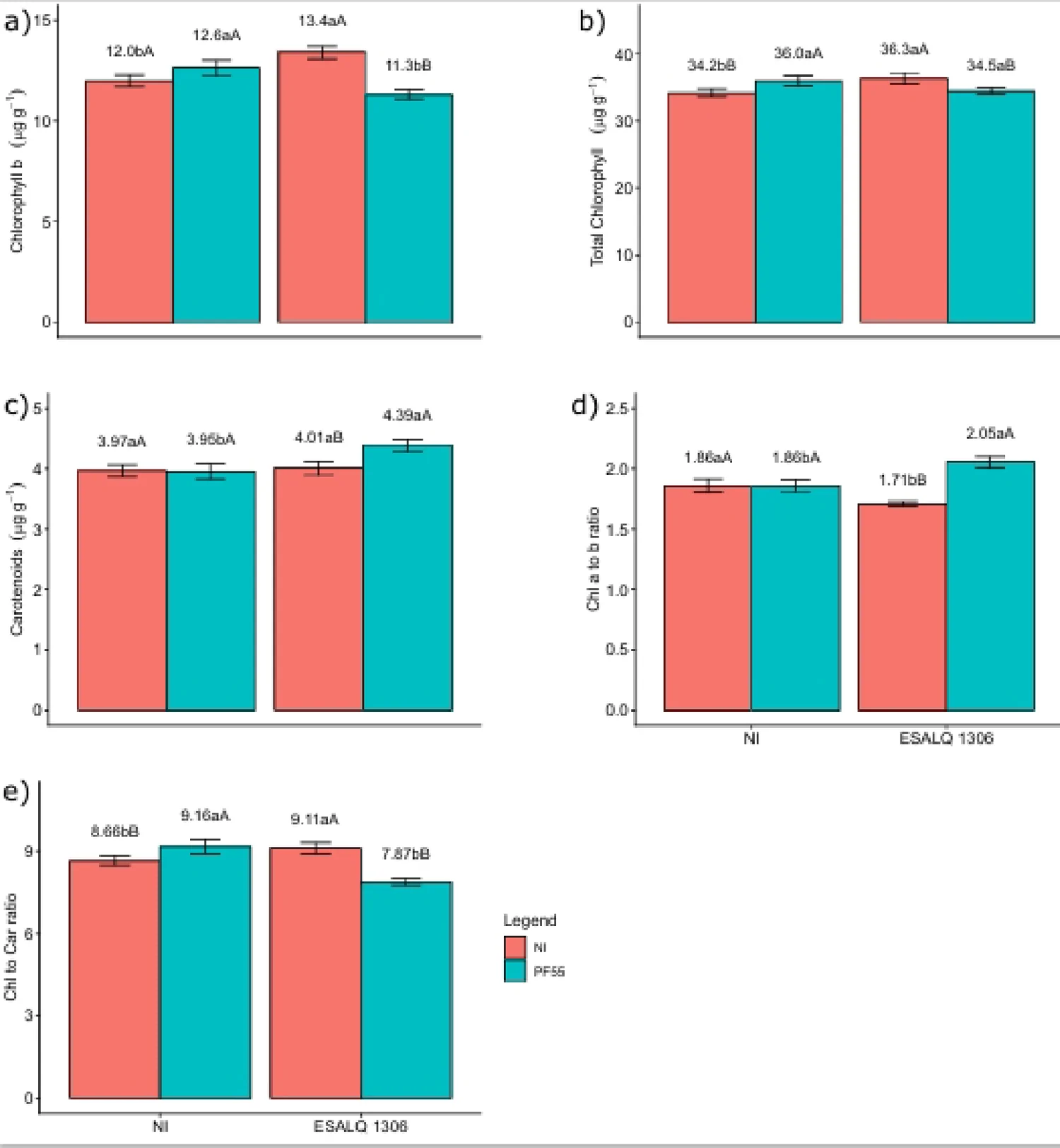
Chlorophyll *b* (**a**), total chlorophyll (**b**), total carotenoids (**c**), total chlorophyll/total carotenoids ratio (**d**), and chlorophyll *a*/chlorophyll *b* ratio (**e**) in onion cultivar Valessul under inoculation with PF55 and ESALQ 1306. Bars represent means ± standard error. Red and blue bars represent the absence (NI) and presence (PF55) of bacterial inoculation, respectively. Different lowercase letters indicate significant differences between ESALQ 1306 levels within each PF55 level (same bar color), whereas different uppercase letters indicate significant differences between PF55 levels within each ESALQ 1306 level (adjacent bars), according to Tukey’s test (*p* < 0.05)

Chlorophyll *a* contents showed no significant differences among treatments. Isolated inoculation with PF55 and ESALQ 1306 increased chlorophyll *b* contents by 11.5% and 18.6%, respectively, relative to coinoculation (Fig. 2a) (*p* < 0.05). The non-inoculated treatment was inferior to inoculation with ESALQ 1306 but did not differ from PF55 inoculation. Regarding total chlorophyll (Fig. 2b), isolated inoculation with PF55 and ESALQ 1306 provided increases of 5.3% and 6.1% compared to the non-inoculated control (*p* < 0.05). However, coinoculation resulted in total chlorophyll content 5.2% lower than that obtained with isolated ESALQ 1306 (Fig. 2b). For total carotenoids (Fig. 2c), coinoculation was superior by 9.5% and 11.1% to treatments inoculated with ESALQ 1306 and PF55 (*p* < 0.05), respectively. In the total chlorophyll/total carotenoids ratio (Fig. 2d), coinoculation was 15.8% and 16.4% lower than treatments with ESALQ 1306 and PF55; the non-inoculated treatment showed similar behavior, also lower than these two treatments. For the chlorophyll a/chlorophyll b ratio (Fig. 2e), coinoculation was higher by 19.9% and 10.2% than treatments with ESALQ 1306 and PF55 (*p* < 0.05), respectively. Additionally, inoculation with ESALQ 1306 promoted an 8.8% reduction compared to the non-inoculated treatment.

**Fig. 3:**
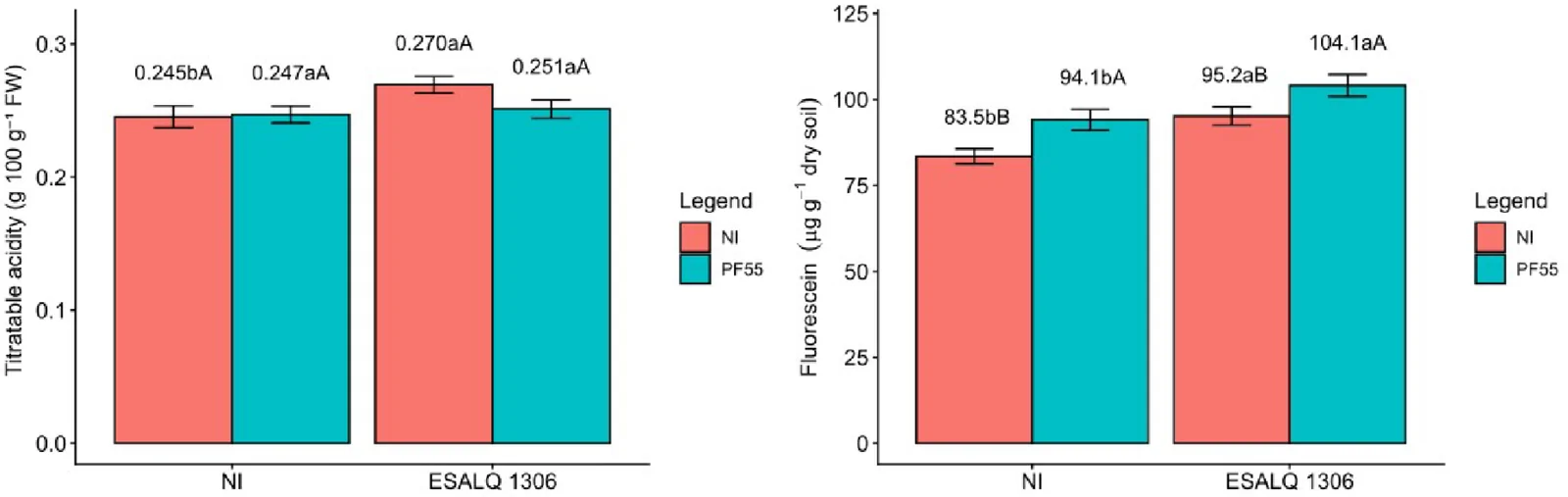
Bulb titratable acidity (**a**) and soil biological activity assessed through fluorescein diacetate hydrolysis (**b**) in onion cultivar Valessul under inoculation with PF55 and ESALQ 1306. Bars represent means ± standard error. Red and blue bars represent the absence (NI) and presence (PF55) of bacterial inoculation, respectively. Different lowercase letters indicate significant differences between ESALQ 1306 levels within each PF55 level (same bar color), whereas different uppercase letters indicate significant differences between PF55 levels within each ESALQ 1306 level (adjacent bars), according to Tukey’s test (*p* < 0.05)

Soluble solids contents (10.4–10.6 °Brix), bulb water content (90.4–91.7%), and pH (5.45–5.59) did not differ among treatments (*p* > 0.05). Titratable acidity, expressed as pyruvic acid equivalents (Fig. 3a), was higher in ESALQ 1306 treatments relative to the non-inoculated control, showing a 10.2% increase (*p* < 0.05). Soil microbial activity, assessed by fluorescein diacetate hydrolysis, showed increases of 10.6% and 9.3% in coinoculation means relative to isolated inoculations with PF55 and ESALQ 1306, respectively (Fig. 3b). The non-inoculated treatment was inferior to treatments with isolated microorganisms.

**Fig. 4:**
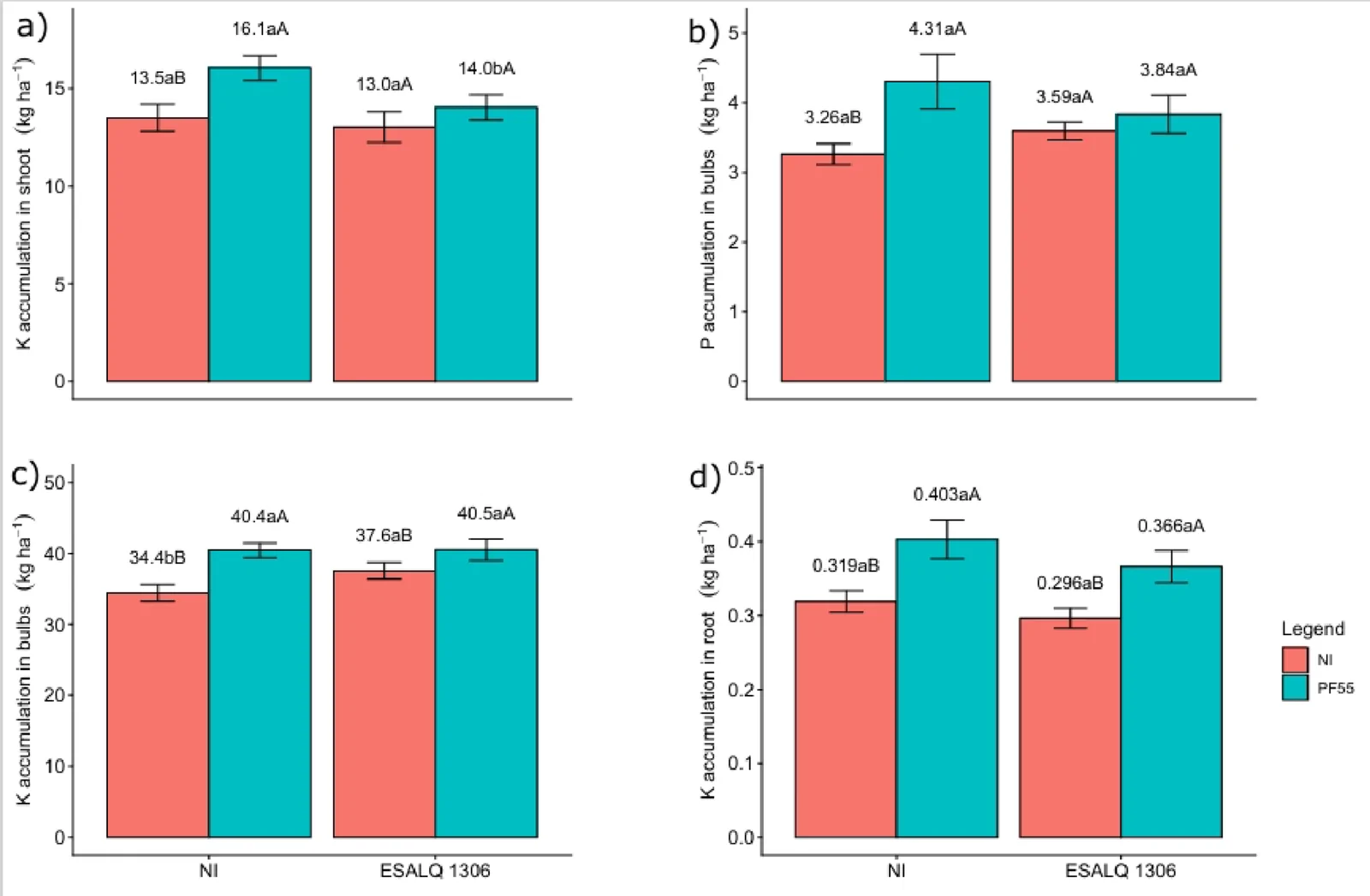
Accumulated potassium in shoots (kg ha⁻¹) (**a**), accumulated phosphorus in bulbs (kg ha⁻¹) (**b**), accumulated potassium in bulbs (kg ha⁻¹) (**c**), and accumulated potassium in the root system (kg ha⁻¹) (**d**) in onion cultivar Valessul under inoculation with PF55 and ESALQ 1306. Bars represent means ± standard error. Red and blue bars represent the absence (NI) and presence (PF55) of bacterial inoculation, respectively. Different lowercase letters indicate significant differences between ESALQ 1306 levels within each PF55 level (same bar color), whereas different uppercase letters indicate significant differences between PF55 levels within each ESALQ 1306 level (adjacent bars), according to Tukey’s test (*p* < 0.05)

Treatments did not influence phosphorus accumulation in shoots or the root system. Potassium accumulation in shoots (Fig. 4a) was higher with isolated PF55 inoculation compared to coinoculation and the non-inoculated treatment, with increases of 14.6% and 19%, respectively (*p* < 0.05). Phosphorus accumulation in bulbs (Fig. 4b) was increased by PF55 inoculation by approximately 32% relative to the non-inoculated treatment (*p* < 0.05). For potassium in bulbs (Fig. 4c), coinoculation was higher than isolated ESALQ 1306 inoculation by approximately 7.8% (*p* < 0.05). The non-inoculated treatment yielded lower values than inoculation with PF55 or ESALQ 1306. The presence of PF55, whether inoculated alone or in combination with ESALQ 1306, led to increased potassium in bulbs (*p* < 0.05). PF55 inoculation increased potassium accumulation in roots (Fig. 4d), regardless of ESALQ 1306 presence. When applied alone, the increase over the non-inoculated treatment was 26.3%; in coinoculation with ESALQ 1306, it was 23.6%.

## Discussion

This study evaluated the effects of inoculating *Pantoea phytobeneficialis* PF55, alone or in association with *Trichoderma harzianum* ESALQ 1306, on productivity, biochemical parameters, and soil microbial activity in onion cv. Valessul. The results indicate that the microorganisms exerted distinct functions in the crop, with the bacterium being determinant for increasing productivity and nutrient accumulation, while the fungus showed predominantly modulating action associated with physiological and edaphic adjustments. This contrast suggests that the mechanism of action of *P. phytobeneficialis* is more directly linked to growth promotion via nutrient solubilization and hormone production (Nascimento et al. 2020), whereas *T. harzianum* acts mainly through physiological modulation and resistance induction (Liriano et al., 2015). These effects do not always translate into biomass gains under field conditions (Contreras-Cornejo et al. 2009). Thus, the absence of productivity increase does not invalidate its role as a physiological and defense modulator but indicates that, under the conditions of this study, the direct effect on yield was limited. Coinoculation, in turn, revealed interactions resulting in specific gains, such as increased carotenoid contents, indicating that the combined effect is not additive for all traits but depends on complex physiological adjustments, in which functional compatibility between isolates is determinant for the success of microbial formulations (Minchev et al. 2021).

Onion productivity was mainly increased by inoculation with *P. phytobeneficialis*, resulting in values superior to the control. This result confirms the bacterium’s potential as a bioinoculant and suggests that growth stimulation is related to its capacity for nitrogen fixation, phosphate solubilization, and phytohormone production (Nascimento et al. 2020). Furthermore, the observed increase indicates that the bacterium’s action impacts multiple physiological processes, from greater root expansion that enhances soil exploration to more efficient nutrient transport to reserve organs. This interpretation is consistent with the higher bulb dry mass recorded in inoculated treatments, demonstrating that effects were not limited to vegetative growth but directly affected the plant’s commercial part. In contrast, T. harzianum applied alone did not promote productivity increase, maintaining values close to the control. This result suggests that, although the fungus is recognized for its biocontrol effect and resistance induction in various crops, its direct contribution to onion productivity increase may be limited under field conditions when applied alone (Metwally et al. 2021).

Similar results were reported by Tanwar et al. (2013) in broccoli (*Brassica oleracea* var. italica), where microbial consortium performance depended strongly on compatibility between associated microorganisms. Coinoculation of *Trichoderma viride* with *Pseudomonas fluorescens* and arbuscular mycorrhizal fungi (*Glomus intraradices* and *Acaulospora laevis*) resulted in higher biomass values, chlorophyll content, and N and P uptake, indicating synergistic effects among agents. On the other hand, less functionally compatible combinations showed reduced performance, reinforcing that consortium compatibility is a critical factor for coinoculation success.

In biomass analysis, bulb and root dry mass increased consistently with bacterial inoculation. The root increase can be attributed to auxin production and other growth regulators by *P. phytobeneficialis*, favoring root expansion and soil exploration (Nascimento et al. 2020), which consequently contributes to greater aerial and bulb biomass stock. The fungus’s presence modulated the bacterium’s effect on shoot dry mass, suggesting lack of additive effect for this parameter. This response may be associated with rhizospheric interaction between microorganisms or differentiated functional action (Yağmur et al. 2024). A similar situation was reported in an experiment with *Trichoderma harzianum* and *Stenotrophomonas* spp., where simultaneous presence of both organisms resulted in values lower than the isolated bacterium treatment for shoot biomass (Singh et al. 2019). Thus, microbial compatibility is a critical factor for determining effective biomass gains, and its understanding is essential for developing more efficient consortiated formulations.

In pigment metabolism, coinoculation showed distinct responses. Chlorophyll a contents remained stable, indicating that this pigment was not affected by microbial interaction. In contrast, chlorophyll b and total chlorophyll contents were reduced in coinoculation relative to isolated treatments, suggesting interference in the synthesis or stability of these pigments. This effect may be related to modulation of metabolic pathways associated with chlorophyll biosynthesis, possibly due to bacterium-fungus interaction (Georgiou and Tsikou 2025; Wang et al. 2021). On the other hand, increased carotenoid contents were observed in coinoculated treatments, indicating activation of photoprotection and antioxidant defense mechanisms. Carotenoids act as dissipators of excess energy and protect photosynthetic membranes and proteins against reactive oxygen species (Han et al. 2025). These pigment variations were accompanied by higher chlorophyll a/b ratio and lower total chlorophyll/carotenoids ratio, reflecting reorganization of photosynthetic complexes with a greater proportion of antioxidant pigments. This pattern is compatible with microorganism-induced responses associated with stress tolerance and defense mechanism activation (Gori et al. 2021; Han et al. 2025).

Bulb sensory quality remained stable with inoculation. Soluble solids and water percentage were constant, in agreement with Altintas and Bal (2008), who reported absence of consistent inoculant effects on these attributes. This result indicates that coinoculation does not substantially alter soluble sugar metabolism or bulb water balance, factors directly related to consumer perception of sweetness and texture (Latt et al. 2025; Kleman et al. 2024). Titratable acidity was slightly higher in treatments inoculated with ESALQ 1306, suggesting activation of organic acid pathways, as reported by Aguiar et al. (2018). This increase may reflect microorganism-mediated adjustments in secondary metabolism, modulating pyruvic and malic acid synthesis, which contribute to flavor and also act as intermediates in defense and cellular respiration processes (Todeschini et al. 2018; Wei et al. 2021). The general stability of sensory attributes is relevant, as it ensures that adopting inoculants, while promoting gains in productivity and physiological characteristics, does not compromise determinants for commercial acceptance of onion in the fresh market.

Soil microbial activity showed a consistent response to inoculation. Coinoculation resulted in the highest FDA hydrolysis values, surpassing the control and isolated treatments. Simultaneous bacterium-fungus interaction can stimulate more complex microbial trophic networks, favoring nutrient cycles and soil functional stability, as described in coinoculation studies (Severo et al. 2025; Wang and Kuzyakov 2024). Stable soil pH levels (5.45–5.59) reinforce that observed changes were biological rather than chemical in nature, an aspect relevant for productive system sustainability (Wang and Kuzyakov 2024; Severo et al. 2025). This result is particularly important in intensive systems, where nitrogen fertilization and recurrent pesticide use can favor soil acidification over productive cycles (Tadesse et al. 2024; Osinuga et al. 2023).

Regarding mineral nutrition, nutrient contents in plant tissues remained constant, while total accumulation was higher in inoculated treatments due to greater plant growth (Li et al. 2023). This pattern reinforces that inoculant efficiency should be interpreted considering the balance between growth and assimilation, as plants with higher biomass accumulate nutrients in greater quantity, even without changes in tissue concentrations (Wang et al. 2023).

For phosphorus, isolated inoculation with *P. phytobeneficialis* increased bulb accumulation, while coinoculation showed performance similar to isolated *T. harzianum*. This pattern indicates that the bacterium exerts a direct effect on P availability, likely through phosphate solubilization and organic acid secretion, mechanisms already described for the species (Nascimento et al. 2020). The non-additive response in coinoculation suggests functional interaction between microorganisms without joint potentiation for this nutrient (Wang and Kuzyakov 2024). For potassium, both isolated inoculation and coinoculation with *P. phytobeneficialis* increased contents in bulbs and roots, surpassing treatment with *T. harzianum* alone and the control. This behavior reinforces that the bacterium was the main agent in improving nutrient absorption and accumulation, while the fungus showed more specific contribution. Overall, the similar behavior between coinoculation and isolated bacterium indicates that P and K gains are essentially attributable to *P. phytobeneficialis* action, reinforcing that functional compatibility of the microbial consortium is determinant for expressing joint effects (Din et al. 2025).

Overall, the results demonstrate that inoculation with *P. phytobeneficialis* PF55 promotes consistent gains in productivity, biomass, and nutrient accumulation, while coinoculation with *T. harzianum* ESALQ 1306 results in specific effects, such as increased carotenoids and soil microbial activity. This pattern confirms that the bacterium plays a central role as a growth promoter (Nascimento et al. 2020), whereas the fungus exerts a modulating function directed to physiological and edaphic processes (Sarma et al. 2025; Metwally et al. 2021; Bononi et al. 2020).

The absence of additive synergism for all evaluated parameters indicates that interactions between microorganisms vary according to the analyzed trait. This behavior is expected, as microbial consortia can show distinct responses depending on plant species, soil resource availability, and isolate adaptation to the environment (Din et al. 2025). In the present study, while microorganism association strengthened photoprotection mechanisms and increased soil biological activity, it did not consistently potentiate productivity and nutrition gains, indicating that consortium choice should be based on careful and targeted evaluations for agronomic objectives.

Although the results were consistent, the study has limitations that must be considered. Evaluations were performed in a single productive cycle under specific soil and climate conditions, which may limit direct extrapolation of results to other environments. Additionally, molecular or enzymatic analyses that could better detail microorganism interactions were not included. Future studies should encompass different edaphoclimatic conditions, cultivation cycles, and complementary molecular and biochemical approaches to deepen understanding of mechanisms determining microbial consortia compatibility and efficiency.

## Conclusion

The inoculation of *P. phytobeneficialis* PF55, alone or in coinoculation with *T. harzianum* ESALQ 1306, positively influenced the agronomic, biochemical, and microbiological performance of the Valessul onion cultivar. The results demonstrated that *P. phytobeneficialis* PF55 was the primary growth-promoting agent, providing increases in productivity, biomass, and nutrient accumulation in plant tissues. In contrast, *T. harzianum* ESALQ 1306 exhibited a modulating effect, contributing in a more restricted manner, especially in elevating soil microbial activity and modifying leaf pigments, mainly chlorophyll b and total chlorophyll.

In summary, coinoculation did not result in additive effects on productivity, but it favored soil biological activity and the physiological balance of the plants, evidencing that interactions between microorganisms are varied and still poorly understood at the field level. Nevertheless, the association between *P. phytobeneficialis* PF55 and *T. harzianum* ESALQ 1306 shows potential to integrate biological management strategies in onion cultivation.

## Data Availability

The datasets generated and/or analyzed during the current study are available from the corresponding author upon reasonable request.

## Abbreviations

BDA: Potato dextrose agar
DMSO: Dimethyl sulfoxide
FDA: Fluorescein diacetate
NPK: Nitrogen-phosphorus-potassium
TSB: Tryptone soy broth
UFC: Colony-forming units

## Materials availability

The microbial strains used in this study are commercially available as part of registered products (Trichodermil^®^ and Benefarm PF55^®^).
